# Mycophenolate mofetil for the treatment of Henoch-Schönlein purpura nephritis; current knowledge and new concepts

**DOI:** 10.15171/jnp.2017.17

**Published:** 2016-12-04

**Authors:** Azar Nickavar, Mahnaz Sadeghian

**Affiliations:** ^1^Department of Pediatric Nephrology, Iran University of Medical Sciences, Tehran, Iran; ^2^Department of Pediatric Gastroenterology, Iran University of Medical Sciences, Tehran, Iran

**Keywords:** Henoch-Schönlein purpura,, Mycophenolate mofetil, Henoch-Schönlein purpura nephritis, Vasculitis

**Implication for health policy/practice/research/medical education:**

Renal involvement is the main predictive factor of long-term outcome in children with Henoch-Schönlein purpura (HSP) . Different immunosuppressive medications have been introduced for the treatment of severe HSP nephritis to prevent subsequent renal impairment. Therapeutic effect of mycophenolate mofetil and its implication have been *discussed in this editorial article. *

Henoch-Schönlein purpura (HSP) is the most common primary systemic vasculitis of childhood (1). It is generally considered a self-limited disease, and majority of patients improve completely (2,3). Short- and long-term outcomes of HSP are generally favorable, and predicted by the severity of initial manifestations and renal involvement (1,2).

Henoch-Schönlein purpura nephritis (HSPN) is the major cause of mortality and morbidity in children with HSP (1), which occurs in 30%-80% of patients during the first three weeks of the initial presentation (1,4).

Majority of patients with HSPN have a mild disease, presenting with hematuria and/or low-grade proteinuria. A small percentage of patients present with nephrotic syndrome or renal impairment (1). However, severe renal involvement is rare in children, compared that in adults (3).

HSPN accounts for 1.8%–3% of children with chronic kidney disease (5), and chronic renal failure might occur in 11%–38% of patients with severe manifestations and pathologic changes (4).

Severity of initial manifestations and histopathologic lesions have been considered the best prognostic factor of HSPN in children (1,6). Accordingly, older age at presentation,‏ hypertension, crescentic glomerulonephritis, significant glomerulosclerosis and tubulointerstitial changes, increased serum creatinine, massive proteinuria, nephrotic or mixed nephritic–nephrotic syndrome have been suggested the major risk factors of future renal impairment (1,2), and imply for aggressive treatment in patients with HSPN (5).

There is no absolute consensus for the best management of severe HSPN and the most effective treatment remains controversial (5-7). Early aggressive immunosuppressive drugs such as cyclosporine, cyclophosphamide, azathioprine, steroid pulse therapy, IV-IgG, rituximab, methotrexate, plasmapheresis, chlorambucil, tacrolimus, and mycophenolate mofetil (MMF) have been suggested for treatment of severe and rapidly progressive HSPN (2,6,8).

There are limited data regarding MMF in children with severe HSPN. It is an immunosuppressive drug, which inhibits B and T cell proliferation, decreases antibody production, prevents glycosylation of adhesion molecules and intercellular adhesion to endothelial cells, inhibits mesangial cell proliferation, and induces apoptosis of activated T cells. It has been reported a safe and effective treatment for inducing and maintaining remission, reducing relapse rate and improving kidney function in frequently relapsing idiopathic nephrotic syndrome, with steroid sparing effect and minimal complication rate (9).

MMF have been successfully used for decreasing massive urine protein excretion in patients with IgA nephropathy. Based on the similar pathologic changes, MMF has been suggested to improve proteinuria and renal function in patients with HSPN (6,10).

There have been some conflicting results about the therapeutic effect of MMF in HSPN. Some studies showed no significant difference in urine protein excretion between MMF and azathioprine treatment. In addition, therapeutic effect of cyclosporine and MMF in children with severe HSPN remained unclear (11).

MMF have been recommended in severe cases of HSPN with nephrotic syndrome, acute nephritis, mixed nephritic/nephrotic syndrome, or steroid resistant nephrotic range proteinuria (6), with steroid sparing effect. It has been a safe and effective treatment for inducing and maintaining stable remission (12), along with improving glomerular cellularity and extracellular matrix deposition in patients with severe HSPN (2).

It has been a valuable and promising alternative agent for the treatment of complicated HSPN with steroid resistance, steroid dependence, and steroid side effects (2,5). Therapeutic effect of MMF associated with low dose prednisone has been as equal as full dose steroid treatment, with more than 70% remission rate (4).

It has been useful for improvement of renal function in severe acute nephritis with decreased glomerular filtration rate (10). Remission rate occurred earlier in MMF treated, and proteinuria improved significantly after the first month of treatment (4). In addition, MMF had less side effects and relapse rate compared to the other immunosuppressive drugs in the majority of studies (1,4,6).

In conclusion, MMF treatment have been recommended for the treatment of severe HSPN, with favorable outcome and low complication rate.

## Authors’ contribution

AN and MS wrote the manuscript equally.

## Conflicts of interest

The authors declared no competing interest.

## Funding/Support

None.
